# Progressive skin necrosis of a huge occipital encephalocele

**DOI:** 10.4103/0970-0358.41120

**Published:** 2008

**Authors:** Yasir Andarabi, Farideh Nejat, Mostafa El-Khashab

**Affiliations:** Department of Neurosurgery, Children′s Medical Center, Tehran University of Medical Sciences, Tehran, Iran; 1 Department of Neurosurgery, Hackensack University Medical Center, Hackensack, New Jersey, USA

**Keywords:** Necrosis, occipital encephalocele, torsion

## Abstract

**Objects:**

Progressive skin necrosis of giant occipital encephalocoele is an extremely rare complication found in neonates. Infection and ulceration of the necrosed skin may lead to meningitis or sepsis. We present here a neonate with giant occipital encephalocoele showing progressive necrosis during the first day of his life.

**Methods:**

A newborn baby was found to have a huge mass in the occipital region, which was covered by normal pink-purplish skin. During the last hours of the first day of his life, the sac started becoming ulcerated accompanied with a rapid color change in the skin, gradually turning darker and then black. The neonate was taken up for urgent excision and repair of the encephalocele. Two years after the operation, he appears to be well-developed without any neurological problems.

**Conclusion:**

Necrosis may have resulted from arterial or venous compromise caused by torsion of the pedicle during delivery or after birth. The high pressure inside the sac associated with the thin skin of the encephalocoele may be another predisposing factor. In view of the risk of ulceration and subsequent infection, urgent surgery of the necrotizing encephalocele is suggested.

## INTRODUCTION

Encephalocoele is a broad term representing a cystic congenital malformation in which central nervous system structures in communication with cerebrospinal fluid (CSF) pathways, herniate through a defect in the cranium. Encephaloceles occur in roughly one out of every 5,000 live births. They may be covered with normal skin, dysplastic skin or a thin, distorted meningeal membrane. Poor prognostic features include large size of the sac, significant brain herniation, abnormality of the underlying brain, microcephaly and ventriculomegaly.[1,2] Hydrocephalus and infection are common complications encountered in the postoperative period. This is a report of a newborn with progressive skin necrosis of large occipital encephalocele.

## CASE HISTORY

**Figure 1:**
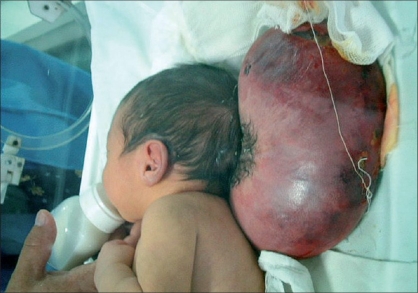
Preoperative photograph of the patient after the start of the skin necrosis. Note the darker and black color change of the skin and the patchy ulcers on the top of the sac with serosanguineous discharge

**Figure 2:**
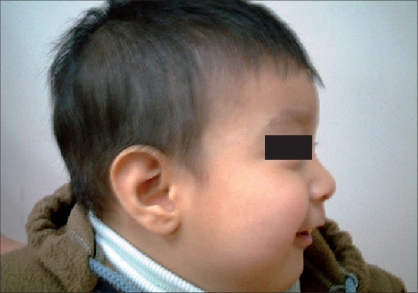
Photograph of the patient at age two with normal appearance

A male neonate was referred from an obstetric hospital two hours after being born through an unplanned, albeit uncomplicated, vaginal delivery. He was the product of an uneventful pregnancy in a 30 year-old mother with three older normal children. The baby had no prenatal diagnosis and cried immediately after birth. There was a huge mass at the occipital region since birth, which was covered by normal pink-purplish skin. The smallest and largest circumferences of the encephalocele were 38 and 60 cm respectively. He was being breast-fed with a good general condition and scheduled for a magnetic resonance imaging (MRI) study of the brain. During the last hours of the first day of his life, the swelling started becoming ulcerated accompanied with a rapid color change in the skin of the sac, gradually turning darker and then black [Figure 1]. The encephalocele had no CSF leakage but the black patchy ulcers on the top of the sac had serosanguineous discharge. The pedicle of this huge sac did not show any evidence of torsion at the base that had a vertical diameter of 6 cm. The child had no evidence of sepsis. The routine hematological and biochemical investigations were normal. He underwent an urgent brain ultrasound study that confirmed a small amount of occipital neural tissue inside the sac without hydrocephalus. In view of the gangrene of the skin and risk of meningitis or sepsis, the neonate was taken up for an urgent excision and repair of the encephalocele. At surgery, the encephalocele was attached to the occipital region via a thin stalk. The neural tissue was resected and complete dural and skin closure was done. The postoperative period was unremarkable. Two years after the operation, he appears to be well-developed without any neurological problems [Figure 2].

## DISCUSSION

Encephalocoeles account for 10 to 20% of all craniospinal dysraphisms. Occipital encephalocoeles are by far the most common type in the Western Hemisphere constituting nearly 85% of all cases, while the Sincipital group predominates in Asia.[3,4] The gender ratio of anterior lesions is roughly equal whereas 70% of occipital encephalocoeles occur in females.[2] The skin covering may be normal or dysplastic.

Prenatal diagnosis of encephalocoeles is possible through ultrasonography, maternal serum Alfa-fetoprotein (MSAFP) and amniocentesis.[4,5]

In the presence of an encephalocele, there is a 60-80% risk of associated structural abnormality both intra-and extra cranially in prenatal series and a 50% risk in postnatal studies. Large occipital encephalocoeles have been found associated with optical, choroidal and retinal dysplasia,[6] severe ocular alterations,[7] central nervous system anomalies,[8] epilepsy,[9] dermoid cyst,[10] tectocerebellar dysraphia[11] and necrosis.[12] More than 60% of these patients may also develop hydrocephalus requiring a ventriculoperitoneal shunt.[2,13,14] Survival rates and morbidity of encephalocoeles vary most strongly with anatomical sites being 100 and 50% respectively in the case of anterior defects and 55 and 83% respectively in the case of posterior defects.[1]

The decision regarding surgery depends on various factors including the amount of neural tissue in the sac, size of the encephalocele, necrosis of the sac, nature of the skin covering and other congenital anomalies.[3,15] Surgical repair of occipital encephalocele can be done electively. However, the presence of CSF leakage or as in our case, necrosis of the skin and risk of subsequent infection warrant a swift decision about surgery.[14]

Skin necrosis of large encephalocoeles is extremely rare. Only one case was found reported in a comprehensive MEDLINE search from 1966 to September 2006. Necrosis may result from hypo perfusion in the microcirculation of the skin of the sac caused by torsion of the pedicle during delivery or after birth. Torsion may be caused because of the large size of the sac having a comparatively small diameter of the pedicle. Starting of necrosis from the distal (uppermost) part of the lesion in this case, might confirm hypo perfusion injury caused by torsion. The other probable cause of necrosis of a large encephalocele of a neonate born through unplanned vaginal delivery is pressure necrosis resulting from the pressurizing of the huge sac against the mothers bony pelvis.[14] Delivery of a newborn with a huge encephalocele is an absolute indication for caesarean section to decrease the chances of complications for both mother and child. As the case was an unplanned delivery, the mother was referred to our obstetric centre just after starting the process of vaginal delivery. In the multiparous mother, he might have had some abnormal positions in the mother′s bony pelvis before or during delivery, increasing chances of compressions on the sac.

## CONCLUSION

Skin necrosis of encephalocoeles is extremely rare. Necrosis may result from hypo perfusion caused by torsion of pedicle during or after delivery. The other cause of necrosis may be pressure necrosis caused by pressurizing of the large sac in the bony pelvis during an unplanned vaginal delivery. In view of the risk of meningitis or sepsis, urgent excision of necrotizing encephalocele is suggested.
